# The impact of 5‐alpha reductase inhibitors on multiparametric MRI diagnostic performance and prostate‐specific antigen density triage: A systematic review

**DOI:** 10.1002/bco2.70294

**Published:** 2026-10-30

**Authors:** Gregory Harrison, Bogdan Toia, Anju Sahdev, Prabhakar Rajan, Benjamin W. Lamb

**Affiliations:** ^1^ Department of Urology Whittington Health NHS Trust London UK; ^2^ Department of Urology University College London Hospitals (UCLH) NHS Foundation Trust London UK; ^3^ Centre for Cancer Cell and Molecular Biology, Barts Cancer Institute Queen Mary University of London London UK; ^4^ Department of Urology Barts Health NHS Trust London UK; ^5^ Department of Urology University College London Hospitals NHS Foundation Trust London UK; ^6^ North East London Cancer Alliance NHS North East London London UK

**Keywords:** 5‐alpha reductase inhibitor, biopsy, diagnostic accuracy, multiparametric MRI, PI‐RADS, prostate cancer, PSA density

## Abstract

**Objectives:**

To evaluate the impact of 5‐alpha reductase inhibitor (5‐ARI) exposure on multiparametric MRI (mpMRI) diagnostic performance for clinically significant prostate cancer (csPCa) and to assess whether standard prostate‐specific antigen density (PSAD)‐gated biopsy thresholds remain safe in this population.

**Subjects/patients and methods:**

A systematic review followed PRISMA 2020 (PROSPERO: CRD420251050507). PubMed/MEDLINE, EMBASE and the Cochrane Library were searched from January 2015 to December 2024. Eligible studies reported PI‐RADS, PSAD or lesion‐level apparent diffusion coefficient (ADC) data by exposure status, biopsy‐verified csPCa (ISUP grade group ≥2) and an unexposed comparator (≥100 patients). Risk of bias was assessed using QUADAS‐2 and ROBINS‐I; heterogeneity precluded meta‐analysis.

**Results:**

Seven comparative studies met inclusion criteria. Patient‐level csPCa diagnostic accuracy was largely maintained: Sensitivity at a PI‐RADS ≥3 threshold was 93.8% in exposed versus 96.1% in unexposed men, csPCa detection was 39.1% versus 39.3% (p = 0.9), and 5‐ARI exposure was not an independent negative predictor of detection. On blinded matched review, peripheral zone lesion ADC was higher in exposed men (869 vs. 765 mm^2^/s, p = 0.04) with lower conspicuity (1.59 vs. 1.78, p = 0.03); that cohort also reported lower adjusted per‐lesion yield (OR 0.32, 95% CI 0.18–0.57), confined to the peripheral zone. A PSAD threshold of >0.15 ng/ml/cc avoided biopsy in 37.0% of exposed versus 30.3% of unexposed men, with missed csPCa in 12.3% and 11.0%, respectively.

**Conclusion:**

Across seven heterogeneous observational studies, patient‐level mpMRI performance for csPCa appears largely preserved in men receiving 5‐ARIs, though this lacks pooled support and was not reproduced in the one lesion‐level, zone‐stratified analysis. Standard PSAD‐gated thresholds, evidenced by a single cohort, may perform comparably across populations. Prospective validation is required.

## 1 INTRODUCTION

Multiparametric magnetic resonance imaging (mpMRI) has restructured the diagnostic pathway for suspected prostate cancer.^1^, ^2^, ^3^, ^4^ The landmark PRECISION trial demonstrated MRI‐targeted biopsy detects more clinically significant prostate cancer (csPCa) and fewer insignificant cancers than non‐targeted transrectal ultrasound (TRUS) biopsy, leading to the adoption of MRI‐first strategies globally.^1^ The PROMIS study independently confirmed mpMRI sensitivity of 88% and negative predictive value of 76% for ISUP grade group ≥2 cancer at a PI‐RADS ≥3 threshold.^2^ Imaging findings are probability‐based for significant prostate cancer and standardised using the Prostate Imaging–Reporting and Data System (PI‐RADS).^3^ PI‐RADS scores 1 and 2 in focal lesions represent a low probability of malignancy, while PI‐RADS 4 and 5 lesions carry a higher probability mandating histological confirmation.^3^, ^4^ The latest European Association of Urology (EAU) guidelines strongly recommend mpMRI both for biopsy‐naïve men and before repeat biopsy when clinical suspicion of significant prostate cancer persists despite previous negative biopsies.^4^

PI‐RADS 3 lesions constitute an equivocal risk where clinical management requires maximising csPCa detection and limiting unnecessary biopsies and associated morbidity.^3^, ^4^, ^5^, ^6^ To navigate this uncertainty, major international guidelines rely on the integration of prostate‐specific antigen density (PSAD) to risk‐stratify patients.^4^ The EAU 2024 guidelines advocate for a structured, two‐tier decision matrix: Biopsy may be safely deferred in favour of surveillance if PSAD is <0.15 ng/ml/cc but is firmly indicated if PSAD ≥0.20 ng/ml/cc.^4^ Similarly, in the United Kingdom, the National Institute for Health and Care Excellence (NICE) endorses a PSAD threshold of >0.15 ng/ml/cc to trigger biopsy, while the operational Getting It Right First Time (GIRFT) framework suggests a more conservative >0.12 ng/ml/cc threshold.^5^, ^6^ Conversely, the American Urological Association (AUA) 2023 guidelines adopt a less prescriptive approach; while they mandate targeted biopsy for PI‐RADS 3–5 lesions, they explicitly recommend incorporating adjunctive clinical metrics, such as a PSAD >0.15 ng/ml/cc, to inform shared decision‐making, particularly in the repeat biopsy setting.^7^

The biomarker stability is disrupted in patients undergoing treatment with 5‐alpha reductase inhibitors (5‐ARIs) such as finasteride and dutasteride.^8^, ^9^, ^10^ Prescribed for benign prostatic disease, these agents induce glandular apoptosis by competitively inhibiting the 5‐alpha reductase enzyme, thereby suppressing intraprostatic dihydrotestosterone (DHT).^9^ Macroscopically, this reduces total prostate volume by approximately 25% and artificially suppresses serum PSA levels by ~50% after 6 months of therapy.^10^ As PSA and prostate volume decline together with 5‐ARI therapy, the net effect on PSAD is not immediately predictable. Proportionate suppression of both parameters could maintain a stable PSAD, whereas disproportionate suppression could shift patients across existing diagnostic thresholds. This uncertainty, rather than an assumed unidirectional effect on PSAD, is the central rationale motivating this review. Historically, large chemoprevention trials, specifically the Prostate Cancer Prevention Trial (PCPT) and REDUCE, demonstrated that 5‐ARIs reduced overall prostate cancer incidence by ~23%–25%, preferentially depleting low‐grade, indolent tumours.^11^, ^12^ Early fears that 5‐ARIs induced high‐grade disease were subsequently attributed to detection and grading artefacts in smaller, 5‐ARI‐atrophied prostates, with long‐term follow‐up of the PCPT demonstrating no associated increase in cancer‐specific mortality.^13^, ^14^

5‐ARIs also induce microscopic functional changes that influence mpMRI radio‐physics.^15^, ^16^, ^17^, ^18^ Standard prostate MRI relies on diffusion‐weighted imaging (DWI) and the apparent diffusion coefficient (ADC), a measure based on Brownian motion that indicates cellular density, membrane density and the microcellular environment for functional cellular evaluation of tissues.^3^, ^15^ Pharmacologically induced glandular atrophy due to 5‐ARI therapy has shown to increase tumour ADC and reduce the conspicuity of focal lesions on DWI.^16^, ^17^ In the MAPPED trial, 6 months of dutasteride produced a mean 36% reduction in T2‐defined lesion volume against a mean 12% increase on placebo (between‐group difference 48%, 95% CI 27.4–68.3; p < 0.0001).^17^ This has led to the proposed, but not yet definitively established, concept of ‘PI‐RADS migration’, a hypothesised mechanism whereby lesions that might otherwise be classified as highly suspicious PI‐RADS 4 or 5 could be functionally downgraded to indeterminate PI‐RADS 3 in exposed men.^16^, ^17^

This systematic review's objective is to evaluate the impact of 5‐ARI exposure on mpMRI diagnostic performance for csPCa and secondarily to assess whether standard PSAD‐gated biopsy thresholds provide safe, equitable triage in this pharmacologically altered population.

## 2 SUBJECTS/PATIENTS AND METHODS

### 2.1 Study design and search strategy

This systematic review was conducted in accordance with the Preferred Reporting Items for Systematic Reviews and Meta‐Analyses (PRISMA) 2020 guidelines.^19^ The review protocol was registered as ‘PROSPERO 2025 CRD420251050507’ prior to screening. A systematic search of PubMed/MEDLINE, EMBASE and the Cochrane Library was performed for literature published between January 2015 and December 2024. Search terms combined the concepts of 5‐ARIs (finasteride, dutasteride), PSA‐related biomarkers (PSA, PSA density, PSAD) and imaging (multiparametric MRI, PI‐RADS), combined with prostate biopsy outcomes.

### 2.2 Study eligibility framework (PICO)

Population: Men undergoing mpMRI for suspected prostate cancer, with a subgroup receiving 5‐ARI therapy for benign prostatic disease.

Intervention/exposure: Documented 5‐ARI exposure at the time of mpMRI, with duration recorded as reported by each study (range 3 months to ≥1 year).

Comparator: Untreated (5‐ARI‐naïve) men undergoing mpMRI within the same cohort.

Outcomes: Primary; mpMRI diagnostic performance for csPCa (ISUP grade group ≥2), measured via PI‐RADS distribution, sensitivity/specificity and lesion‐level ADC. Secondary; performance of PSAD‐gated biopsy thresholds (0.10–0.20 ng/ml/cc) in avoiding unnecessary biopsy without missing csPCa.

### 2.3 Eligibility and study selection

Included studies comprised real‐world diagnostic registries and clinical cohorts evaluating mpMRI performance in men with suspected prostate cancer. The study selection process is summarised in the PRISMA 2020 flow diagram (Figure S1). Strict inclusion criteria required:

- Documented mpMRI utilising PI‐RADS v2 or v2.1
- Explicit reporting of PSAD, lesion‐level ADC values, or PI‐RADS distribution by 5‐ARI exposure status
- Biopsy verification reporting csPCa (ISUP grade group ≥2)
- An explicit comparison between a 5‐ARI exposed cohort and an unexposed control arm, with a total study cohort of >100 patients

The initial database search identified 961 records. Following screening at the title and abstract level, 938 records were excluded (primarily non‐prostate MRI studies, non‐original research, basic science/dermatology reports or studies lacking any 5‐ARI exposure). We sought and retrieved the remaining 23 full‐text articles for detailed eligibility assessment. Of these, 16 were excluded with specific reasons: Eleven lacked a dedicated non‐exposed comparative cohort, two were narrative reviews/meta‐analyses, two featured inadequate sample sizes (n < 100), and one evaluated quantitative T2‐imaging without utilising a standard PI‐RADS v2/v2.1 scoring protocol.

Ultimately, seven core comparative studies met all strict predefined criteria and were included in the final analysis. The registered protocol specified a minimum age of 40 years; this criterion was not explicitly applied during screening, as age was not consistently reported at the cohort level across candidate studies.

### 2.4 Data extraction and quality assessment

Given the volume of the initial yield (961 records), a pragmatic screening approach was adopted. One author (G.H.) screened all titles and abstracts, and a second author (B.T.) independently screened a random 20% sample of abstracts to assess agreement. All 23 retrieved full‐text articles were then independently assessed by both authors. A third reviewer (B.L.) was available to adjudicate discrepancies but was not required.

Data extraction captured 5‐ARI exposure duration, MRI field strength and biopsy approach. Continuous variables were harmonised for reporting. To preserve functional fidelity, ADC values were extracted exactly as reported on a lesional basis. Risk of bias was evaluated using QUADAS‐2 for the two cohorts appraised primarily for diagnostic accuracy and ROBINS‐I for the observational comparative cohorts^20^, ^21^; domain‐level judgements appear in Table 6, with full assessments in Appendices A and B. As the instruments interrogate different domains, judgements are not treated as equivalent across them. This was different from the planned structure for meta‐analysis registered in the PROSPERO, and therefore, the review was classified as a systematic review.

### 2.5 Data synthesis and protocol deviations

The protocol specified quantitative synthesis, but clinical and methodological heterogeneity precluded meta‐analysis. Exposure definitions ranged from 3 months to more than 1 year, biopsy pathways differed, and one study analysed lesions rather than patients. Findings are therefore presented as a structured narrative synthesis with tabulated effect estimates.

The registered protocol also specified independent dual screening of the full dataset, with a process to resolve differences; in practice, dual abstract screening was applied to a random 20% sample only, with full‐text eligibility subsequently assessed independently by two authors. The authorship group felt this was appropriate due to the large number of studies and adjustment from meta‐analysis to systematic review. The registered protocol additionally proposed a head‐to‐head comparative evaluation of NICE (>0.15 ng/ml/cc) versus GIRFT (>0.12 ng/ml/cc) PSAD thresholds and a dedicated analysis of ADC thresholds for diagnostic accuracy in 5‐ARI users; the final review reports PSAD threshold performance descriptively via decision curve analysis but does not formally compare guideline thresholds head‐to‐head and does not derive or validate a specific ADC cut‐off, reflecting the limited granularity of ADC and threshold‐specific data available across the included observational cohorts.

## 3 RESULTS

### 3.1 Study selection and characteristics

Following systematic application of the PRISMA 2020 framework, seven core studies met strict inclusion criteria.^19^, ^22^, ^23^, ^24^, ^25^, ^26^, ^27^, ^28^ These comprised five patient‐level comparative diagnostic cohorts (Falagario et al., Kim et al., Aksu et al., Morote et al., Wang et al.), one single‐centre consecutive MRI‐targeted biopsy cohort (Savin et al.) and one dedicated matched lesion‐level MRI analysis (Das et al.).^22^, ^23^, ^24^, ^25^, ^26^, ^27^, ^28^ Study designs and primary endpoints are detailed in Table 1.

**TABLE 1:** Characteristics of the seven included studies.

Panel A. Study designs, exposure definitions and primary endpoints						
Study	Design and setting	Exposure definition	Exposed/unexposed	Unit	Biopsy pathway	Primary endpoint
Falagario et al.^22^	Retrospective multicentre PROMOD registry, 24 institutions	≥3 months at MRI	705/6913 patients	Patient	Systematic biopsy if MRI‐negative; targeted plus systematic if MRI‐positive	Diagnostic accuracy and PSAD decision curve analysis
Kim et al.^24^	Single‐centre retrospective fusion‐biopsy cohort	>1 year at biopsy	80/626 patients	Patient	TRUS/MRI fusion‐targeted plus systematic biopsy	csPCa detection and multivariable predictors
Aksu et al.^23^	Single‐centre retrospective cognitive‐targeting cohort	5‐ARI use as reported	70/359 patients	Patient	Transrectal cognitive targeting; PI‐RADS ≥3 enrolled	PI‐RADS distribution and overall malignancy
Morote et al.^28^	Retrospective case–control across 10 centres, Catalonia	>1 year	120/2092 full; 118/118 matched	Patient	Targeted and/or 12‐core systematic biopsy	PI‐RADS distribution and csPCa detection
Wang et al.^26^	Single‐centre retrospective cohort	Documented use: duration subgroups reported	54/297 patients	Patient	Transperineal saturation biopsy ± fusion targeting	Cancer detection and extended biomarkers
Savin et al.^25^	Single‐centre retrospective consecutive cohort	≥6 months before MRI; dutasteride only	52/548 patients	Patient	Combined transperineal fusion‐targeted and systematic biopsy	Lesion features and targeted biopsy outcomes
Das et al.^27^	Retrospective multi‐institutional lesion‐level cohort with blinded matched imaging sub‐study	≥3 months before MRI; median 41 months	90 /1018 lesions; 60/658 patients	Lesion	MRI–TRUS fusion‐targeted biopsy; 1:1 genetically matched blinded review	Zone‐specific csPCa detection, lesion ADC and conspicuity

Panel B. Baseline demographic and biomarker characteristics						
Study	Summary statistic	Age, years	Prostate volume, ml	PSA, ng/mL	PSAD, ng/ml/cc	Notes
Falagario^22^	Median (IQR)	69/66	53/50	6.0/6.5	0.11/0.13	PSA not corrected in exposed men
Kim^24^	Median (IQR)	69.0/65.0	41.0/38.7	5.56/7.23	0.13/0.18	Doubled exposed PSA 11.12 ng/ml
Aksu^23^	As published	68.66/66.57	59.9/60.2	5.37/7.12	0.10/0.14	Volume difference not significant (p = 0.835)
Morote,^28^ full	Median (IQR)	72/68	69/53	8.0/7.3	0.12/0.14	PSA p = 0.107; volume p < 0.001; PSAD p = 0.025
Morote,^28^ matched	Median (IQR)	72/71	67/66	8.0/7.5	Not reported	Balanced by 1:1 matching
Wang^26^	Mean ± SD	67.6/65.6	64.9/48.1	11.0/9.41	0.194/0.214	Corrected exposed PSA 22.0; corrected PSAD 0.387. PHI 35.2 versus 47.8 and PHI density 0.667 versus 1.18; both p < 0.01
Savin^25^	Median (IQR)	72/68	60/54	6.8/6.6	Not reported	Previous biopsy 69% versus 47% (p = 0.004)
Das,^27^ lesions	Median (IQR)	70/65	60/49	8/ 8 raw; 16/8 corrected	Not reported	Lesion‐level cohort (90/1018 lesions)

*Note*: This table comprises two panels presented on consecutive pages: Panel A, study designs and endpoints; Panel B, baseline demographic and biomarker characteristics. Das et al. is the only included lesion‐level study with zonal stratification and blinded imaging review; lesion‐level and patient‐level denominators are not interchangeable. Values are exposed/unexposed. Data reported as median where indicated, otherwise mean; medians and means are not treated as interchangeable. Morote et al. balanced baseline characteristics via 1:1 matching, and Das et al. reported lesion‐level characteristics; both are shown with that qualification. Corrected PSA values are identified where applicable. PHI density values are reported as mean ± SD in the source.

Abbreviation: PHI, Prostate Health Index.

### 3.2 Risk of bias within studies

Domain‐level risk‐of‐bias judgements are presented in Table 6. Under QUADAS‐2, both cohorts appraised for diagnostic accuracy carried concerns. Falagario et al. was rated high risk on the index test domain because no centralised blinded radiological review was performed across the 24 contributing institutions. Aksu et al. raised concerns regarding reference‐standard ascertainment because all patients underwent cognitive TRUS‐targeted biopsy, with attendant potential for sampling error, and was rated moderate on the index test domain owing to reliance on a single reader. Under ROBINS‐I, Kim et al. and Morote et al. were judged at low overall risk, Savin et al. moderate, and Das et al. low to moderate, the latter reflecting outcome ascertainment and selective reporting in the dataset available at the time of appraisal. Wang et al. was the only cohort judged at serious overall risk, driven by serious residual confounding, and this judgement is carried forward in the interpretation of its lower overall detection rate.^20^, ^21^, ^23^, ^24^, ^25^, ^26^, ^27^, ^28^

### 3.3 Patient demographics and baseline biomarkers

Demographic and biomarker characteristics, detailed in Table 1, Panel B, reflected the anticipated pharmacodynamic effects of 5‐ARIs, primarily alterations in prostate volume and PSA kinetics.^10^, ^18^, ^22^, ^23^, ^24^, ^25^, ^26^, ^28^ Uncorrected PSA was consistently lower or comparable in treated cohorts, whereas the application of correction factors yielded significantly higher corrected PSA and PSAD values in exposed men.^10^, ^26^ Wang et al. additionally reported significantly lower Prostate Health Index (PHI) values (35.2 vs. 47.8) and PHI density (0.667 vs. 1.18) in exposed men, both p < 0.01.^26^

### 3.4 mpMRI PI‐RADS distributions and migration

Evidence from the primary cohorts demonstrates that 5‐ARI exposure does not cause systematic downgrading of all lesions into the indeterminate PI‐RADS 3 category within the larger, fusion‐guided cohorts, although localised distributional shifts are observed within PI‐RADS categories.^22^, ^24^, ^25^, ^26^, ^28^ Kim et al., Wang et al., Savin et al. and Morote et al.'s matched pairs all found no statistically significant difference in overall PI‐RADS distribution between groups.^24^, ^25^, ^26^, ^28^

In contrast, Aksu et al. observed a statistically significant inflation of PI‐RADS 3 lesions in the 5‐ARI‐exposed group (37.1% vs. 20.6%, p = 0.009).^23^ The Falagario et al. multicentre cohort detected a statistically significant distribution shift globally (p < 0.0001); absolute percentage differences across individual PI‐RADS categories ranged from 1.9 to 7.3 percentage points, the largest being an excess of PI‐RADS 1–2 assignments in treated men (20.0% vs. 12.7%); the per‐category differences are set out in the Table 2 footnote.^22^ Table 2 details these distributions.

**TABLE 2:** PI‐RADS category distribution by exposure status.

Study	Group	PI‐RADS 1–2	PI‐RADS 3	PI‐RADS 4	PI‐RADS 5	Overall p
Falagario	Exposed	141 (20.0%)	158 (22.4%)	258 (36.6%)	148 (21.0%)	p < 0.0001
	Unexposed	878 (12.7%)	1764 (25.5%)	2948 (42.6%)	1323 (19.1%)	
Kim	Exposed	22 (27.5%)	30 (37.5%)	28 (35.1%)^a^	—	p = 0.083
	Unexposed	151 (24.2%)	216 (34.5%)	259 (41.4%)^a^	—	
Aksu^b^	Exposed	n/a	26 (37.1%)	26 (37.1%)	18 (25.7%)	p = 0.009
	Unexposed	n/a	74 (20.6%)	150 (41.8%)	135 (37.6%)	
Morote, full	Exposed	15 (12.5%)	20 (16.7%)	57 (47.5%)	28 (23.6%)	p = 0.328^c^
	Unexposed	320 (14.5%)	429 (20.5%)	903 (43.2%)	440 (21.0%)	
Morote, matched	Exposed	15	20	56	27	p = 0.685
	Unexposed	18	14	56	30	
Wang	Exposed	3 (5.6%)	23 (42.6%)	21 (38.9%)	7 (13.0%)	Not significant
	Unexposed	27 (9.1%)	88 (29.6%)	145 (48.8%)	37 (12.5%)	
Savin^b^	Exposed	n/a	15 (29%)	28 (54%)	9 (17%)	p = 0.69
	Unexposed	n/a	130 (24%)	308 (56%)	110 (20%)	
Das, all lesions^b^	Exposed	n/a	18 (20%)	44 (49%)	28 (31%)	p = 0.01
	Unexposed	n/a	365 (36%)	407 (40%)	246 (24%)	
Das, matched lesions^b^	Exposed	n/a	Not numerically reported^d^	Not numerically reported^d^	Not numerically reported^d^	Not reported^d^
	Unexposed	n/a	Not numerically reported^d^	Not numerically reported^d^	Not numerically reported^d^	

*Note*: Falagario et al. per‐category absolute differences, exposed versus unexposed: 7.3 percentage points for PI‐RADS 1–2 (20.0% vs. 12.7%); 3.1 percentage points for PI‐RADS 3 (22.4% vs. 25.5%); 6.0 percentage points for PI‐RADS 4 (36.6% vs. 42.6%); and 1.9 percentage points for PI‐RADS 5 (21.0% vs. 19.1%). The overall distribution differed significantly (p < 0.0001) in a cohort of 7618 patients.

^a^ Kim et al. categorised suspicious lesions as PI‐RADS 2, 3, 4 or 5 and reported PI‐RADS ≥4 combined.

^b^ Aksu et al., Savin et al. and Das et al. enrolled PI‐RADS ≥3 lesions only.

^c^ Morote et al. Table 1 reports p = 0.328 whereas the accompanying narrative reports p = 0.238; both are recorded without inference. Morote matched‐group percentages in the source are row percentages and are therefore omitted.

^d^ PI‐RADS v2.1 score was included among the genetic‐matching variables used by Das et al. (with age, corrected PSA and prostate volume); category‐level matched counts and a formal between‐group p‐value were not reported in the accessible main text and were not independently reconstructed.

In the Das et al. lesion‐level cohort, the distributional shift ran in the opposite direction, with PI‐RADS four to five lesions accounting for 80% of lesions in exposed men and 64% in unexposed men (p = 0.01).^27^ PI‐RADS v2.1 score was one of four variables used in their genetic matching algorithm, alongside age, corrected PSA and prostate volume, so the matched sub‐cohort would be expected to be balanced on PI‐RADS; category‐level counts and a formal between‐group comparison were not reported in the accessible main text and have not been reconstructed here. Within the matched cohort, lesion location was similar between groups (61% vs. 57% peripheral zone, p = 0.1). The direction of any distributional shift is therefore inconsistent across studies rather than uniformly downward.

### 3.5 Diagnostic performance, csPCa yield and radio‐physics

Diagnostic accuracy of mpMRI for csPCa in 5‐ARI‐exposed men is largely retained at patient level.^22^, ^24^, ^25^, ^26^, ^28^ In the Falagario et al. PROMOD cohort, testing revealed no significant interaction between 5‐ARI exposure and PI‐RADS scoring; sensitivity for csPCa at a PI‐RADS ≥3 threshold was high in both groups (93.84%, 95% CI 90.32–96.37, in treated men vs. 96.06%, 95% CI 95.26–96.76, in untreated men), with corresponding specificities of 28.90% and 18.38%. csPCa was detected in 276/705 (39.1%) exposed and 2718/6913 (39.3%) unexposed men (p = 0.9), although GG ≥ 3 disease was more frequent among exposed men (160/705, 22.7%, vs. 1301/6913, 18.8%; p = 0.013).^22^ Raw biopsy outcomes are presented in Table 3, adjusted and model‐based estimates in Table 4, category‐specific detection in Table S1, and diagnostic accuracy estimates in Table S3.

**TABLE 3:** Biopsy outcomes: raw denominators and unadjusted comparisons.

Study	Outcome	Exposed n/N (%)	Unexposed n/N (%)	Reported p	Unit and endpoint
Falagario	csPCa (GG ≥ 2)	276/705 (39.1%)	2718/6913 (39.3%)	p = 0.9	Patient; combined biopsy
Falagario	GG ≥ 3	160/705 (22.7%)	1301/6913 (18.8%)	p = 0.013	Patient; combined biopsy
Kim	Any cancer	29/80 (36.3%)	228/626 (36.4%)	p = 0.976	Patient; total cohort
Kim	csPCa	23/80 (28.8%)	152/626 (24.3%)	p = 0.169	Patient; total cohort
Kim	csPCa, MRI‐positive	21/58 (36.2%)	137/475 (28.8%)	p = 0.074	MRI‐positive subgroup
Aksu	Overall malignancy	33/70 (47.1%)	174/359 (48.5%)	p = 0.505	Patient; csPCa not reported
Morote, full	Any PCa	66/120 (55.0%)	1336/2092 (63.9%)	p = 0.052	Patient
Morote, full	csPCa	51/120 (42.5%)	935/2092 (44.7%)	p = 0.706	Patient
Morote, full	Insignificant PCa	15/120 (12.5%)	401/2092 (19.2%)	p = 0.072	Patient
Morote, matched	Any PCa	65/118 (55.1%)^a^	70/118 (59.3%)^a^	p = 0.599	Source reports row % 48.1/51.9
Morote, matched	csPCa	50/118 (42.4%)^a^	45/118 (38.1%)^a^	p = 0.596	Source reports row % 52.6/47.4
Morote, matched	Insignificant PCa	15/118 (12.7%)^a^	25/118 (21.2%)^a^	p = 0.089	Source reports row % 37.5/62.5
Wang	Any PCa	25/54 (46.3%)	202/297 (68.0%)	p < 0.01	Patient; saturation ± targeted
Wang	csPCa	17/54 (31.5%)	135/297 (45.5%)	p = 0.0566	Patient
Wang	csPCa among detected cancers	17/25 (68.0%)	135/202 (66.8%)	p = 0.907	Conditional denominator
Savin	Targeted csPCa	16/52 (31%)	166/548 (30%)	p = 1.00	Targeted cores only
Savin	Overall csPCa	23/52 (44%)	188/548 (34%)	p = 0.17	Combined targeted and systematic
Savin	Any cancer	30/52 (57%)	353/548 (64%)	p = 0.45	Combined biopsy
Savin	Insignificant cancer	7/52 (13%)	165/548 (30%)	p = 0.01	Combined biopsy
Das	csPCa, peripheral zone lesions	29%	39%	p = 0.008 (unexposed PZ vs. TZ)	Lesion‐level; percentages as published in source
Das	csPCa, transition zone lesions	28%	31%	p = 0.9 (exposed PZ vs. TZ)	Lesion‐level; numerators not reported

^a^ Within‐group proportion recalculated from the published numerator and denominator; published row percentages are shown in the final column. Outcomes include clinically significant prostate cancer (csPCa, ISUP grade group ≥2), grade group ≥3 disease, any prostate cancer, insignificant cancer and overall malignancy, as reported by each study. No adjusted or model‐based estimates appear in this table (see Table 4). Patient‐level and lesion‐level estimates are not pooled. Aksu et al. did not report a csPCa‐specific outcome. Das et al. reported zonal detection percentages without numerators; the p‐values shown are the within‐group zonal comparisons as published, not exposed versus unexposed tests.

Abbreviation: GG, ISUP grade group.

**TABLE 4:** Adjusted, matched and model‐based estimates.

Study	Analysis type	Outcome	OR (95% CI)	p‐value	Adjustment and qualification
Falagario	Multivariable logistic regression	csPCa	1.05 (0.60–1.84)	0.876	Age, biopsy history, DRE, PSA, MRI volume, PI‐RADS, exposure
Falagario	PI‐RADS × 5‐ARI interaction	PI‐RADS 3	1.00 (0.49–2.03)	0.996	Same model
Falagario	PI‐RADS × 5‐ARI interaction	PI‐RADS 4	1.04 (0.56–1.96)	0.892	Same model
Falagario	PI‐RADS × 5‐ARI interaction	PI‐RADS 5	1.33 (0.65–2.69)	0.437	Same model
Kim	Multivariable logistic regression	csPCa	1.258 (0.750–2.111)	0.384	Age, PSA, prostate volume, PI‐RADS, exposure
Morote	Multivariable logistic regression	csPCa	0.826 (0.521–1.311)	0.418	Age, PSA, DRE, biopsy type, family history, volume, PI‐RADS
Morote	Multivariable logistic regression	Insignificant PCa	0.628 (0.357–1.104)	0.106	Same covariates
Savin	Univariate logistic regression	Targeted csPCa	1.02 (0.5–1.9)	0.94	Unadjusted
Savin	Univariate logistic regression	PI‐RADS ≥4 score	0.76 (0.4–1.4)	0.41	Unadjusted
Das	Multivariable logistic regression	Lesion‐level csPCa	0.32 (0.18–0.57)	<0.001	Age, corrected PSA, volume, PI‐RADS v2.1, active surveillance
Das	PI‐RADS‐adjusted model	Lesion‐level csPCa	0.53 (0.31–0.87)	0.014	PI‐RADS score alone
Das	Genetically matched model	Lesion‐level csPCa	0.41 (0.24–0.68)	0.03	Matched on age, corrected PSA, volume, PI‐RADS v2.1
Das	Matched zonal model	Peripheral zone csPCa	0.20 (0.05–0.68)	0.016	Matched cohort
Das	Matched zonal model	Transition zone csPCa	0.38 (0.11–1.18)	0.11	Matched cohort

*Note*: An odds ratio below 1 indicates lower odds of detection with 5‐ARI exposure. Savin et al. reported univariate estimates only, which are not comparable with the adjusted models. Das et al. estimates are lesion‐level and are not directly comparable with the patient‐level models.

Kim et al. and Savin et al. found that 5‐ARI use was not an independent negative predictor for csPCa detection on targeted biopsy.^24^, ^25^ Kim et al. reported csPCa in 23/80 (28.8%) exposed and 152/626 (24.3%) unexposed men (p = 0.169), with an adjusted odds ratio for exposure of 1.258 (95% CI 0.750–2.111, p = 0.384).^24^ Savin et al. reported targeted csPCa in 16/52 (31%) versus 166/548 (30%) (p = 1.00) and, on combined targeted and systematic sampling, overall csPCa in 23/52 (44%) versus 188/548 (34%) (p = 0.17); these are distinct biopsy endpoints and are reported separately throughout. Their regression estimates were univariate (exposure and targeted csPCa, OR 1.02, 95% CI 0.5–1.9, p = 0.94) and are labelled as such in Table 4. Insignificant cancer was less frequent among exposed men in that cohort (7/52, 13%, vs. 165/548, 30%; p = 0.01), who were also older and more often previously biopsied (69% vs. 47%, p = 0.004).^25^

Wang et al. noted a lower overall cancer detection rate in the 5‐ARI group (25/54, 46.3%, vs. 202/297, 68.0%; p < 0.01), which aligns with the known chemo‐preventive effect of 5‐ARIs selectively depleting low‐grade tumours, although this cohort carried the only serious ROBINS‐I confounding judgement in the review.^11^, ^12^, ^26^ Morote et al. found no significant difference in csPCa detection in their full cohort (51/120, 42.5%, vs. 935/2092, 44.7%; p = 0.706). Their matched results were published as row percentages; recalculated as within‐group proportions these give csPCa in 50/118 (42.4%) exposed and 45/118 (38.1%) unexposed men (p = 0.596), with insignificant cancer in 15/118 (12.7%) and 25/118 (21.2%), respectively (p = 0.089).^28^ Aksu et al. reported overall malignant pathology only (33/70, 47.1%, vs. 174/359, 48.5%; p = 0.505), and their results are therefore not treated as csPCa estimates.^23^

Das et al. was the only included study to report a statistically significant reduction in csPCa detection with 5‐ARI exposure. In the full 1108‐lesion cohort, the association persisted after adjustment for age, corrected PSA, prostate volume, PI‐RADS score and active‐surveillance status (adjusted OR 0.32, 95% CI 0.18–0.57, p < 0.001), was reproduced in a 1:1 genetically matched cohort (marginal OR 0.41, 95% CI 0.24–0.68, p = 0.03), and in zone‐specific analysis was present for peripheral zone lesions (OR 0.20, 95% CI 0.05–0.68, p = 0.016) but not transition zone lesions (OR 0.38, 95% CI 0.11–1.18, p = 0.11).^27^

The functional radio‐physics underlying these observations were examined in the blinded matched imaging sub‐study of Das et al., in which 37 exposed and 32 unexposed peripheral zone lesions and 28 exposed and 32 unexposed transition zone lesions were re‐reviewed. Median peripheral zone lesion ADC was higher in exposed men (869 vs. 765 mm^2^/s, p = 0.04), while background peripheral zone ADC did not differ (1328 vs. 1376 mm^2^/s, p = 0.2), and lesion conspicuity, defined as the ratio of surrounding‐zone to lesion ADC, was lower (1.59 vs. 1.78, p = 0.03). No corresponding differences were demonstrated in the transition zone, where lesion ADC (780 vs. 811 mm^2^/s, p = 0.13), background ADC (1066 vs. 1182 mm^2^/s, p = 0.07) and conspicuity (1.36 vs. 1.38, p = 0.7) were all comparable. Full values with interquartile ranges are given in Table 5. This zonal pattern is directionally consistent with the randomised MAPPED data.^16^, ^17^, ^27^

**TABLE 5:** Zonal lesion ADC and conspicuity on blinded matched imaging review (Das et al.).

Zone	Imaging characteristic	5‐ARI use, median (IQR)	No 5‐ARI use, median (IQR)	p‐value	Interpretation
Peripheral zone	Lesion ADC, mm^2^/s	869 (706–978)	765 (646–844)	0.04	Higher lesion ADC in exposed men
Peripheral zone	Background zone ADC, mm^2^/s	1328 (1208–1472)	1376 (1296–1512)	0.2	No difference in adjacent non‐tumour tissue
Peripheral zone	Lesion conspicuity	1.59 (1.37–1.87)	1.78 (1.63–2.12)	0.03	Lower conspicuity in exposed men
Transition zone	Lesion ADC, mm^2^/s	780 (673–858)	811 (711–966)	0.13	No significant difference
Transition zone	Background zone ADC, mm^2^/s	1066 (1050–1152)	1182 (1067–1228)	0.07	Non‐significant trend
Transition zone	Lesion conspicuity	1.36 (1.25–1.68)	1.38 (1.23–1.59)	0.7	No significant difference

*Note*: Values are medians with interquartile ranges from the blinded matched imaging sub‐study of Das et al., comprising 37 exposed and 32 unexposed peripheral zone lesions and 28 exposed and 32 unexposed transition zone lesions. Lesion conspicuity is defined as the ratio of surrounding‐zone ADC to lesion ADC, so lower values indicate a less conspicuous lesion. Wilcoxon rank sum and rank sum exact tests. These are the only lesion‐level radio‐physics data available across the included studies; no other cohort reported zonal ADC by exposure status.

Abbreviation: ADC, apparent diffusion coefficient.

These accuracy figures require cautious interpretation. In several cohorts, the decision to biopsy was informed by the mpMRI result, and men with negative imaging were less likely to undergo biopsy. Without a reference standard independent of the index test, csPCa missed in both groups may not have been ascertained, so the reported sensitivity and negative predictive values may be overestimates.

### 3.6 Decision curve analysis and PSAD triage strategies

Extrapolating previously published decision curve analysis data from the Falagario et al. cohort demonstrates the clinical efficiency of gating biopsy decisions behind standardised PSAD thresholds. Due to the pharmacologically altered baseline characteristics of 5‐ARI users, applying standard PSAD thresholds systematically diverts a larger percentage of 5‐ARI users away from biopsy. Utilising the NICE threshold of >0.15 ng/ml/cc for PI‐RADS 3 lesions avoids 261/705 biopsies (37.0%) in exposed men compared with 2093/6913 (30.3%) in unexposed men; all 10 reported strategies appear in Table S2. The corresponding proportions of missed csPCa were 34/276 (12.3%) in exposed men and 300/2718 (11.0%) in unexposed men.^22^ These estimates derive from a single cohort and have not been independently replicated.^5^, ^6^, ^7^, ^22^

## 4 DISCUSSION

The clinical uncertainty surrounding PI‐RADS scoring in men with 5‐ARI‐induced glandular change can now be examined against a comparative evidence base, albeit one that is discordant.^10^, ^16^, ^17^, ^18^ Across seven comparative imaging studies, patient‐level mpMRI diagnostic accuracy for csPCa was largely preserved, although the one study to analyse individual lesions with zonal stratification reached the opposite conclusion. 5‐ARI exposure did not significantly alter the diagnostic performance of PI‐RADS scoring, with sensitivity maintained at 94% in treated men at the PI‐RADS ≥3 threshold, suggesting that the standard imaging pathway is not substantially compromised in this population. This conclusion is drawn from observational cohorts without pooled quantitative synthesis and should be interpreted accordingly.^22^, ^23^, ^24^, ^25^, ^26^, ^27^, ^28^

That said, 5‐ARI exposure produces a measurable subclinical effect on imaging. Peripheral zone ADC values are significantly elevated in exposed men compared to unexposed controls (869 vs. 765 mm^2^/s, p = 0.04; Table 5), a finding that offers a plausible, though unconfirmed, mechanistic contribution to the ‘PI‐RADS migration’ phenomenon reported in targeted cohorts.^16^, ^17^, ^23^, ^27^ Decision curve analysis derived from a single large cohort suggests that standard PSAD thresholds (0.15–0.20 ng/ml/cc) may achieve comparable biopsy‐avoidance across exposed and unexposed populations, although this finding requires confirmation in independent datasets before it can be considered generalisable.^4^, ^5^, ^6^, ^7^, ^22^

The hypothesis that 5‐ARI exposure significantly degrades mpMRI diagnostic performance is not supported by multivariable and interaction analyses from large cohorts.^22^, ^24^, ^25^ Falagario et al. found no evidence that 5‐ARI exposure modifies the association between PI‐RADS score and csPCa.^22^ These findings are supported by Kim et al. and Savin et al., in which 5‐ARI exposure was not an independent negative predictor of csPCa detection after accounting for PI‐RADS score.^24^, ^25^ Interestingly, Wang et al. found that overall cancer detection rates were significantly lower in 5‐ARI users.^26^ This lower yield mirrors the chemoprevention of low‐grade cancers observed in the PCPT and REDUCE trials.^11^, ^12^, ^13^, ^14^ However, Wang et al. carries the highest ROBINS‐I confounding risk of the included studies, reflecting its small 5‐ARI sample (n = 54), single‐centre design and use of saturation biopsy rather than MRI‐fusion guidance. As this is a different sampling strategy, it limited direct comparability to the fusion‐guided cohorts. The reduction in overall detection was not replicated at the level of csPCa specifically (31.5% vs. 45.5%, p = 0.057), and csPCa‐to‐total‐cancer ratios were preserved between groups (68.0% vs. 66.8%, p = 0.907). This reinforces a chemo‐preventive rather than diagnostic explanation for the lower yield. Despite functional shifts, standard biopsy triggers for highly suspicious PI‐RADS 4 and 5 lesions remain unaltered.^22^, ^24^, ^25^, ^26^, ^28^

The discordance between Das et al. and the patient‐level cohorts is the central interpretive problem of this review. Das et al. analysed individual lesions and stratified by prostate zone, whereas Falagario et al. reported patient‐level outcomes from a 24‐institution registry; a zone‐specific effect may be diluted where peripheral and transition zone lesions are not separated. The zonal data in Table 5 offer a coherent mechanism for that dilution, since the imaging changes were demonstrable only in the peripheral zone. Conversely, the multicentre composition of the Falagario cohort, without centralised radiological review, may better reflect routine practice while introducing inter‐reader variation, and it was rated high risk on the QUADAS‐2 index test domain for that reason. Differences in biopsy pathway, exposure‐duration definition and PSA correction further restrict comparison. Neither unqualified reassurance nor a recommendation to modify the pathway is supported.^22^, ^27^

Aksu et al. reported a statistically significant inflation of PI‐RADS 3 lesions in 5‐ARI‐exposed men, whereas the fusion‐guided cohorts did not.^22^, ^23^, ^24^, ^25^, ^26^, ^28^ One plausible explanation is the functional shift in diffusion imaging reported by Das et al., in which peripheral zone lesions in men receiving 5‐ARIs showed higher ADC values than matched unexposed lesions.^27^ This 5‐ARI‐associated increase in ADC is consistent with reduced diffusion restriction and may reduce DWI lesion conspicuity, potentially shifting some PI‐RADS 4 and 5 lesions towards borderline PI‐RADS 3 categorisation.^16^, ^17^, ^27^

Aksu et al. used cognitive TRUS biopsy targeting rather than MRI‐fusion guidance, which introduces greater sampling error and limits the comparability of their distributional finding to fusion‐guided cohorts.^23^ Nevertheless, while overall gland distributions resist systematic downgrading in the largest cohorts, Falagario et al. demonstrated that 10.9% of csPCa cases in treated men were visible only as PI‐RADS 3 lesions, underscoring the importance of robust PSAD‐based triage in this cohort. The absolute magnitude of the PI‐RADS distribution shift observed in the Falagario cohort was modest relative to its statistical significance. This discrepancy is attributable to the very large sample size (n = 7618) affording high power to detect small differences. Therefore, this holds greater clinical weight on this finding relative to studies with smaller, non‐significant shifts.^22^

Long‐term chemoprevention data underpins the convention of doubling PSA after 3–6 months of 5‐ARI therapy.^10^, ^11^, ^12^, ^13^ Morote et al. found no statistically significant difference in raw PSA levels between 5‐ARI users and naïve men at the point of referral (8.0 vs. 7.3 ng/ml, p = 0.107), suggesting that in real‐world clinical practice, men are often referred when PSA is rising rather than at a pharmacologically suppressed baseline.^28^ Despite heterogeneity in PSA handling, PSAD appears highly robust as a triage parameter because both PSA and volume are affected in opposite directions, naturally stabilising the density metric at the point of MRI referral.^10^, ^18^, ^22^, ^26^, ^28^

Decision curve analysis from the Falagario cohort suggests that PSAD‐gated rules may divert a larger fraction of 5‐ARI users away from unnecessary biopsies without an apparent increase in missed csPCa, though this finding awaits confirmation beyond a single dataset. Based on currently available evidence, there is no clear indication for a separate biopsy algorithm in 5‐ARI‐exposed men,^22^ although this should not be regarded as a definitive recommendation given the limitations outlined above. Applying standard PSAD thresholds (0.15–0.20 ng/ml/cc) to PI‐RADS 3 lesions appears to provide broadly comparable cancer‐detection efficiency across populations. As with national guidelines generally, the trade‐off between missed csPCa and avoided biopsies will vary across clinical services.^5^, ^6^, ^7^, ^22^

## 5 LIMITATIONS

The interpretation of our findings must be carefully contextualised. The exclusive reliance on retrospective observational cohorts introduces inherent selection bias and precludes evaluation of longitudinal, within‐subject MRI appearances before and after 5‐ARI initiation.^22^, ^23^, ^24^, ^25^, ^26^, ^27^, ^28^ The ROBINS‐I evaluation, summarised in Table 6, highlighted significant heterogeneity in the handling of baseline clinical variables.^21^, ^22^, ^23^, ^24^, ^25^, ^26^, ^27^, ^28^ Patients prescribed 5‐ARIs consistently present with larger baseline prostate volumes which influence PSA kinetics. Therefore, observational studies that failed to implement statistical matching or multivariable regression exposed their comparative findings to confounding bias.^10^, ^18^, ^22^, ^23^, ^24^, ^25^, ^26^, ^28^ Furthermore, the lack of centralised, blinded radiological review across the largest multicentre datasets introduces an unavoidable degree of inter‐observer variability.^22^, ^28^

**TABLE 6:** Risk of bias assessment.

Panel A. QUADAS‐2 (cohorts appraised for diagnostic accuracy)					
Study	Patient selection	Index test	Reference standard	Flow and timing	Overall judgement
Falagario et al.^22^	Moderate—retrospective design; both biopsy‐naive and biopsy‐experienced patients included	High—no centralised blinded radiological review across 24 institutions	Low—standardised ISUP grading applied throughout	Low—pre‐biopsy MRI in all patients; appropriate sequence	High concern on the index test domain
Aksu et al.^23^	Moderate—retrospective design; high clinical suspicion population	Moderate—single experienced reader; potential reader bias	High—ascertainment concern: cognitive TRUS targeting with attendant sampling error	Low—appropriate imaging‐to‐biopsy timing	High concern on the reference standard domain

Panel B. ROBINS‐I (observational comparative cohorts)						
Study	Selection of participants	Confounding	Outcome measurement	Selective reporting	Classification of exposure	Overall judgement
Kim et al.^24^	Moderate	Low	Low	Low	Low	Low
Savin et al.^25^	Moderate	Moderate	Low	Low	Low	Moderate
Wang et al.^26^	Moderate	Serious	Low	Low	Moderate	Serious
Das et al.^27^	Low	Low	Moderate	Moderate	Low	Low to moderate
Morote et al.^28^	Low	Low	Low	Low	Low	Low

*Note*: In Panel A, the QUADAS‐2 reference standard domain concerns whether the reference standard correctly classifies the target condition and whether its interpretation was influenced by the index test. The concern recorded for Aksu et al. relates to ascertainment, in that all patients underwent cognitive TRUS‐targeted biopsy with attendant potential for sampling error; this is a verification and ascertainment concern rather than a judgement on grading accuracy or blinding. In Panel B, QUADAS‐2 was applied to the two cohorts appraised primarily for diagnostic accuracy and ROBINS‐I to the observational comparative cohorts. The two instruments interrogate different constructs; judgements are not interchangeable between them and no composite score has been calculated across instruments. Full domain‐level assessments with supporting notes are provided in Appendices A and B. Wang et al. was the only cohort judged at serious overall risk, and this judgement is carried into the interpretation of its lower overall detection rate.

Verification bias is a further constraint: Several cohorts were assembled from men who proceeded to biopsy after mpMRI, so MRI‐negative disease may have gone unascertained in both exposure groups. The blinded matched re‐review within Das et al. mitigates reader bias for the radio‐physics outcomes but rests on 69 peripheral zone and 60 transition zone lesions, so the findings in Table 5 are correspondingly imprecise.^22^, ^27^

The review deviated from the registered PROSPERO protocol by abandoning the planned RCT‐based meta‐analysis in favour of an observational, narrative synthesis. This reflects the unanticipated absence of randomised evidence in this population, while methodologically justified given the heterogeneity encountered (biopsy platform, exposure duration, PSA correction methods). This precluded pooled effect estimates and limited the precision of any single conclusion.

Only 20% of abstracts underwent independent duplicate screening; although the primary screener adopted a deliberately over‐inclusive approach to preserve sensitivity, undetected selection bias at this stage cannot be excluded. 5‐ARI exposure duration varied from 3 months to over 1 year across included cohorts, despite pharmacological evidence that maximal glandular suppression requires 6–12 months; pooling short‐ and long‐exposure cohorts may obscure a duration‐dependent effect and could explain some of the discordance between studies (e.g., Aksu et al. vs. the larger fusion‐guided cohorts).^8^, ^9^, ^11^, ^12^ Several of the most influential clinical conclusions, particularly those relating to PSAD‐gated decision curve analysis, derive predominantly from a single large cohort (Falagario et al.) and should not be generalised as consistently replicated findings. No included study was a randomised trial, precluding causal inference; residual confounding by baseline prostate volume, which independently affects PSA kinetics, cannot be fully excluded despite ROBINS‐I‐guided appraisal. Finally, the absence of centralised, blinded radiological review across the largest multicentre datasets introduces inter‐observer variability that may affect PI‐RADS distribution comparisons.

## 6 CONCLUSION

Across seven heterogeneous observational studies, patient‐level mpMRI diagnostic accuracy for csPCa appears largely preserved in men receiving 5‐ARIs, although this conclusion is not supported by pooled quantitative synthesis and is not reproduced in the single lesion‐level, zone‐stratified cohort, which found reduced csPCa detection in peripheral zone lesions.^22^, ^23^, ^24^, ^25^, ^26^, ^27^, ^28^ 5‐ARI exposure is associated with pharmacological effects that elevate peripheral zone lesion ADC values and may occasionally reduce lesion conspicuity, but available evidence does not indicate a consistent, clinically meaningful reduction in patient‐level PI‐RADS diagnostic accuracy.^22^, ^23^, ^24^, ^25^, ^26^, ^27^, ^28^ For equivocal PI‐RADS 3 lesions, existing data, derived predominantly from a single large cohort, suggest that a unified PSAD‐gated triage strategy may perform comparably regardless of exposure status; prospective, multicentre validation is needed before this can be considered an established recommendation.^5^, ^6^, ^7^, ^22^

Prospective, multicentre studies with prespecified zone‐specific analysis, adequate verification of MRI‐negative men and centralised blinded radiological review are required to resolve this discordance.

## AUTHOR CONTRIBUTIONS

**Gregory Harrison:** Literature review; paper identification; data collection; data analysis; manuscript writing. **Bogdan Toia:** Screening of papers for review. **Anju Sahdev:** Paper review and advice. **Prabhakar Rajan:** Paper review and advice. **Benjamin W. Lamb:** Project concept; paper review and advice; third‐party support for literature choice.

## CONFLICT OF INTEREST STATEMENT

Authors above declare no conflict of interest.

## Supporting information


**Figure S1.** PRISMA 2020 study selection flow diagram.
**Table S1.** csPCa detection by PI‐RADS category.
**Table S2.** PSAD‐gated biopsy strategies and biopsy‐triage outcomes reported by Falagario et al.
**Table S3.** Diagnostic accuracy of mpMRI for clinically significant prostate cancer according to 5‐ARI exposure in Falagario et al.

## Data Availability

All data analysed in this review were extracted from previously published studies, which are cited in the reference list.
