# Supplementary material for: The impact of 5‐alpha reductase inhibitors on multiparametric MRI diagnostic performance and prostate‐specific antigen density triage: A systematic review
Source: BJUI Compass. 2026 Oct 30;7(11):e70294. doi: 10.1002/bco2.70294 (PMC13722573; doi:10.1002/bco2.70294)
Supplement: Supplementary file 1 [file BCO2-7-e70294-s001.docx]

**Supporting Information**

*The Impact of 5-Alpha Reductase Inhibitors on Multiparametric MRI Diagnostic Performance and Prostate-Specific Antigen Density Triage: A Systematic Review*

Contents:

- Figure S1, PRISMA 2020 flow diagram.
- Table S1, csPCa detection by PI-RADS category (data extracted from Falagario et al. Supplementary Table 4 and the other source articles).
- Table S2, PSAD-gated biopsy strategies reported by Falagario et al.
- Table S3, diagnostic accuracy of mpMRI for csPCa by 5-ARI exposure in Falagario et al.

*Note: Tables S1 to S3 are this review's own supplementary tables and are numbered independently of the identically numbered supplementary tables of Falagario et al. and Das et al.*

**Figure S1. PRISMA 2020 study selection flow diagram**

*Record counts and exclusion reasons as reported in the Methods.*

**Table S1. csPCa detection by PI-RADS category**

| **Study** | **PI-RADS** | **Exposed n/N (%)** | **Unexposed n/N (%)** | **Source-reported p-value** | **Endpoint and qualification** |
| --- | --- | --- | --- | --- | --- |
| Falagario | 1–2 | 17/141 (12.1%)† | 107/878 (12.2%)† | Not reported for csPCa‡ | csPCa; recalculated within category |
| Falagario | 3 | 30/158 (19.0%)† | 358/1,764 (20.3%)† | Not reported for csPCa‡ | csPCa; recalculated within category |
| Falagario | 4 | 114/258 (44.2%)† | 1,282/2,948 (43.5%)† | Not reported for csPCa‡ | csPCa; recalculated within category |
| Falagario | 5 | 115/148 (77.7%)† | 971/1,323 (73.4%)† | Not reported for csPCa‡ | csPCa; recalculated within category |
| Morote, matched | ≤2 | 0/15 (0%) | 3/18 (16.7%) | p=0.233 | csPCa |
| Morote, matched | 3 | 2/20 (10.0%) | 3/14 (21.4%) | p=0.627 | csPCa |
| Morote, matched | 4 | 26/56 (46.4%) | 16/56 (28.6%) | p=0.078 | csPCa |
| Morote, matched | 5 | 22/27 (81.5%) | 23/30 (76.7%) | p=0.751 | csPCa |
| Wang | 1–2 | 0/3 (0%) | 4/27 (14.8%) | Not reported | csPCa |
| Wang | 3 | 2/23 (8.7%) | 18/88 (20.4%) | Not reported | csPCa |
| Wang | 4 | 10/21 (47.6%) | 82/145 (56.6%) | Not reported | csPCa |
| Wang | 5 | 5/7 (71.4%) | 31/37 (83.8%) | Not reported | csPCa |
| Savin | 3 | 1/15 (6%) | 8/130 (6%) | p=1.00 | Targeted csPCa |
| Savin | 4 | 7/28 (25%) | 87/308 (28%) | p=0.82 | Targeted csPCa |
| Savin | 5 | 8/9 (89%) | 71/110 (65%) | p=0.27 | Targeted csPCa |
| Aksu | 3 | — | — | p=0.105 | Overall malignancy 36.5% vs 19.3% |
| Aksu | 4 | — | — | p=0.031 | Overall malignancy 32.0% vs 53.9% |
| Aksu | 5 | — | — | p=0.597 | Overall malignancy 71.9% vs 77.8% |
| Kim | All categories | Not reported | Not reported | All p>0.05 | csPCa numerators not reported |
| Das | 3–5 | Not reported numerically | Not reported numerically | Source Figure 1A | Lesion-level; figure not digitised |

*†Within-category csPCa detection proportion recalculated using csPCa counts from Falagario et al. Supplementary Table 4 and category denominators from Table 1 of that article. The source supplementary table reports the distribution of PI-RADS categories among csPCa cases, rather than category-specific detection proportions. No values were read off figures.*

*‡Falagario et al. reported category-specific p-values for overall prostate cancer detection (PI-RADS 1–2, p=0.2; PI-RADS 3, p=0.9; PI-RADS 4, p=0.8; PI-RADS 5, p=0.002), not for the recalculated csPCa proportions shown here. These overall-cancer p-values are therefore given in this footnote only and should not be read as formal comparisons of the adjacent csPCa values.*

*Aksu et al. reported overall malignancy only. Data extracted from Falagario et al. Supplementary Table 4 and from the main text and tables of the remaining source articles.*

**Table S2. PSAD-gated biopsy strategies and biopsy-triage outcomes reported by Falagario et al.**

| **Strategy (biopsy if…)** | **Unexposed: avoided, n (%)** | **Unexposed: GG 1 missed** | **Unexposed: GG ≥2 missed** | **Exposed: avoided, n (%)** | **Exposed: GG 1 missed** | **Exposed: GG ≥2 missed** |
| --- | --- | --- | --- | --- | --- | --- |
| Reference: biopsy all PI-RADS 3–5 | 878 (12.7) | 126 (9.7) | 107 (3.9) | 141 (20.0) | 12 (10.5) | 17 (6.2) |
| 1: PI-RADS 4–5 or PSAD >0.20 | 2,252 (32.6) | 414 (32.0) | 340 (12.5) | 265 (37.6) | 40 (35.1) | 38 (13.8) |
| 2: PI-RADS 4–5 or PSAD >0.15 | 1,847 (26.7) | 320 (24.7) | 254 (9.3) | 233 (33.0) | 36 (31.6) | 29 (10.5) |
| 3: PI-RADS 4–5 or PSAD >0.10 | 1,073 (15.5) | 173 (13.4) | 116 (4.3) | 163 (23.1) | 27 (23.7) | 18 (6.5) |
| 4: PI-RADS 3–5 or PSAD >0.20 | 764 (11.1) | 100 (7.7) | 83 (3.1) | 125 (17.1) | 12 (10.5) | 14 (5.1) |
| 5: PI-RADS 3–5 or PSAD >0.15 | 632 (9.1) | 76 (5.9) | 61 (2.2) | 113 (16.0) | 11 (9.6) | 12 (4.3) |
| 6: PI-RADS 3–5 or PSAD >0.10 | 381 (5.5) | 45 (3.5) | 30 (1.1) | 77 (10.9) | 8 (7.0) | 7 (2.5) |
| 7: PI-RADS 4–5, or 3 if PSAD >0.20 | 2,366 (34.2) | 440 (34.0) | 364 (13.4) | 281 (39.9) | 40 (35.1) | 41 (14.9) |
| 8: PI-RADS 4–5, or 3 if PSAD >0.15 | 2,093 (30.3) | 370 (28.6) | 300 (11.0) | 261 (37.0) | 37 (32.5) | 34 (12.3) |
| 9: PI-RADS 4–5, or 3 if PSAD >0.10 | 1,570 (22.7) | 254 (19.6) | 193 (7.1) | 227 (32.2) | 31 (27.2) | 28 (10.1) |
| 10: PI-RADS 4–5, or 3 if PSAD >0.10 or >0.20 | 1,456 (21.1) | 228 (17.6) | 169 (6.2) | 211 (29.9) | 31 (27.2) | 25 (9.1) |

*Data extracted from Falagario et al. Supplementary Table 5. PSAD, prostate-specific antigen density (ng/mL/cc); GG, ISUP grade group. The missed-disease columns refer to grade group ≥2 (clinically significant) and grade group 1 (insignificant) cancer respectively, not to grade group ≥3.*

*Percentages for biopsies avoided use the full exposure-group denominator (705 exposed and 6,913 unexposed patients); percentages for missed GG 1 and GG ≥2 cancers use the corresponding number of cancers of that grade within each exposure group. Proportions in the same row therefore have different denominators and are not directly comparable with one another.*

*Biopsy avoidance was consistently higher in exposed men with broadly similar proportions of missed grade group ≥2 disease. These estimates derive from a single cohort.*

**Table S3. Diagnostic accuracy of mpMRI for clinically significant prostate cancer according to 5-ARI exposure in Falagario et al.**

| **MRI positivity threshold** | **Measure** | **5-ARI treated, % (95% CI)** | **5-ARI untreated, % (95% CI)** |
| --- | --- | --- | --- |
| **Positive MRI defined as PI-RADS ≥3** |  |  |  |
|  | Sensitivity | 93.84 (90.32–96.37) | 96.06 (95.26–96.76) |
|  | Specificity | 28.90 (24.66–33.45) | 18.38 (17.22–19.58) |
|  | Positive predictive value* | 45.92 (44.25–47.60) | 43.26 (42.87–43.66) |
|  | Negative predictive value* | 87.94 (81.81–92.21) | 87.81 (85.55–89.76) |
|  | Accuracy* | 54.33 (50.57–58.05) | 48.92 (47.74–50.11) |
| **Positive MRI defined as PI-RADS ≥4** |  |  |  |
|  | Sensitivity | 82.97 (78.01–87.21) | 82.89 (81.42–84.29) |
|  | Specificity | 58.74 (53.92–63.44) | 51.90 (50.37–53.42) |
|  | Positive predictive value* | 56.40 (53.31–59.45) | 52.75 (51.86–53.64) |
|  | Negative predictive value* | 84.28 (80.33–87.56) | 82.40 (81.09–83.64) |
|  | Accuracy* | 68.23 (64.65–71.65) | 64.08 (62.94–65.21) |

*Data extracted from Falagario et al. Supplementary Table 3. Clinically significant prostate cancer defined as ISUP grade group ≥2. Denominators are 705 treated and 6,913 untreated patients.*

**Positive predictive value, negative predictive value and accuracy are dependent on disease prevalence, as noted in the source table, and are not directly transferable to populations with different csPCa prevalence.*

*These estimates derive from a single multicentre cohort in which the decision to biopsy was informed by the MRI result; sensitivity and negative predictive value are therefore susceptible to verification bias and may be overestimates. No other included study reported sensitivity or specificity by exposure status.*
